# Assessing the quality of life and subjective ratings of the anatomy-based fitting map among experienced, adult cochlear implant users

**DOI:** 10.1016/j.bjorl.2026.101807

**Published:** 2026-03-28

**Authors:** Pelden Wangchuk, Cila Umat, Chong Foong Yen, Faizah Mohd Zaki, Asma Abdullah

**Affiliations:** a Universiti Kebangsaan Malaysia, Faculty of Health Sciences, Center for Rehabilitation and Special Needs Studies, Kuala Lumpur, Malaysia; b JDWNR Hospital, Department of Otorhinolaryngology, Audiology Unit, MoH, Thimphu, Bhutan; c Universiti Kebangsaan Malaysia, Faculty of Medicine, Department of Radiology, Kuala Lumpur, Malaysia; d Universiti Kebangsaan Malaysia, Faculty of Medicine, Department of Otorhinolaryngology-Head and Neck Surgery, Kuala Lumpur, Malaysia

**Keywords:** Anatomy-based fitting, Cochlear implant, Quality of life, Subjective rating

## Abstract

**Objective:**

Anatomy-Based Fitting (ABF) is a relatively new cochlear implant fitting method launched to minimize frequency-to-place mismatch. This study aimed to assess the benefits of using the ABF map among experienced adult Cochlear Implant (CI) users regarding their subjective experiences and Quality of Life.

**Methods:**

This prospective observational study compared the outcome measures between the ABF and the regular Conventional-Based Fitting (CBF) CI maps. Using selective sampling, nine ears were examined from eight experienced adult ME-DEL cochlear implant users. All participants underwent postoperative computed tomography scans, which were then analyzed using a surgical planning software called Otoplan® to determine the positions of intracochlear electrodes, their respective angular insertions, and cochlear duct length. The information from the Otoplan® was then imported into the Maestro 9.0 fitting software to generate the ABF map. The outcome measures compared were (i) Subjective ratings of the maps concerning speech understanding, listening comfort, and preferred map; and (ii) Cochlear implant Quality of Life – 35-items (CIQOL-35). The CBF map’s outcome assessment was completed before fitting the experimental ABF map. The review assessments for ABF were taken after six months of ABF use. Finally, their attitude towards the ABF map was obtained.

**Results:**

The shifts of the ABF center frequencies varied largely concerning the CBF map across participants, ranging from 0.46 semitones to 23.94 semitones. Subjectively, all three domains were rated better for the ABF than the CBF maps (p < 0.05). Overall, the CIQOL-35 scores significantly improved after using the ABF map for six months, with the entertainment domain showing a significant increase in the mean difference (p < 0.05). 87.5% of participants wanted to continue using the ABF map, indicating a positive attitude towards the ABF map.

**Conclusion:**

Results suggest that the ABF map could be an alternative to improve auditory experience and other QoL determinants.

## Highlights

• Anatomy-based fitting is relatively a new cochlear implant mapping technique.
• Frequency-to-place mismatch is addressed by Anatomy-based fitting.
• Quality of life improved after using an anatomy-based fitting method for 6-months.
• Enhanced subjective feedback.
• Improved vowel recognition in quiet is strongly linked with improved enjoyment of entertainment domain in CIQoL-35.

## Introduction

Current literature on image-guided tonotopic fitting aims to address place-pitch-related issues in the Cochlear Implant (CI) signal coding strategies. This study aimed to compare the Quality of Life (QoL) and subjective ratings between image-guided maps, which are known as the Anatomy-Based Fitting (ABF) method, and the standard Conventional-Based Fitting (CBF) among experienced adult CI users.

The Greenwood equation provides the frequency position function of the cochlea, where the sound frequencies correlate to the spatial location of the receptor cells in the organ of Corti.^1^ Signal processing algorithms in the CI processor follow cochlear tonotopic organization.^2^ Therefore, this phenomenon depends on the cochlear coverage obtained by the electrode array of the CI. Cochlear coverage and electrode insertion angles are determined by interindividual anatomical variations in cochlear size, cochlear duct length,^3^, ^4^, ^5^ and different electrode arrays.^6^ This leads to a mismatch between the frequency information programmed by the CI processor versus the acoustical tonotopic characteristic of nerves being excited by intracochlear electrode contact points. This phenomenon is commonly known as a Frequency-to-Place Mismatch (FPM).^7^, ^8^, ^9^ A study by Sturm et.al.^10^ has shown the negative impact of FPM on the quality of life of CI users. A study by Wilson^11^ highlighted the possibilities of improving the outcomes from current CI devices by increasing the spatial specificity of neural excitation with specific electrodes. In addition, recently developed CI surgical planning software with a special focus (Otoplan®, CAScination AG, in collaboration with MED-EL, Innsbruck, Austria) models a three-dimensional representation of the cochlea using Computed Tomography (CT) images, enabling the determination of the position and characteristics of the intracochlear electrode array. The MED-EL Maestro CI fitting software can use the information to execute the map with frequency bands based on the Greenwood function and intracochlear electrode contacts. This fitting method is called Anatomy-Based Fitting (ABF). The programming technique aims to improve the place-pitch matching for a more accurate pitch perception using frequency allocation through imaging information. A recent study of Lassaletta et.al.^12^ had also shared their study experiences with the ABF maps having a lower frequency-to-place mismatch than default fitting maps.

Currently, only a few studies have explored the benefits of ABF. Di Maro and team^13^ experimented with the ABF map for 10 MED-EL post-lingual adult CI users for an average of 41.6-days, and the study revealed better speech perception thresholds in quiet. Similarly, Kurz. et.al.,^14^ found that speech reception in quiet and in noise improved with the ABF map compared to the standard conventional map after three months post-ABF, while assessing with the Oldenburg Matrix test. Similarly, Wangchuk et.al.,^15^ found that improved speech detection with the ABF over CBF maps could be due to accuracy in frequency-to-place mismatch.

Current literature on image-guided tonotopic fitting seems a promising strategy for addressing FPM. The study highlighted the necessity of more data to corroborate the benefit of the ABF over default fitting in speech and subjective tests.^12^ This study aimed to compare outcome measures of the ABF maps as compared to CBF maps among experienced adult CI users, concerning the following outcome measures: (i) Subjective ratings (speech understanding, listening comfort, and map preference); and (ii) Quality of life (QoL) using the Cochlear Implant Quality of Life-35 items (CIQOL-35) instrument by McRackan et al.^16^

We hypothesized that the subjective and QoL ratings would improve after using the ABF map for six months.

## Methods

### Ethics

This study complies with ethical principles and has been approved by the University Research Ethical Committee. A written informed consent was obtained from all participants after providing detailed orientation about the study.

### Participants

A selective sampling was applied to recruit experienced adult MED-EL CI users who were mentally stable to ensure reliable responses. The informed consent was obtained after carefully addressing the following inclusion criteria:

i) Adult CI users (18-years and above).
ii) Using MED-EL CI system with at least Sonnet or Rondo 2 speech processor.
iii) Had at least six months of CI experience to ensure adequate habituation with the electric hearing.
iv) Aided hearing sensitivity within 20–40 dB HL between 250 to 4000 Hz octave frequencies on the CI side.
v) Informally, cognition screening and mentally sound participants through case history to ensure reliable and consistent responses.
vi) Able to speak and understand Malay or English.

Exclusion criteria include: (i) Cochlea malformation as confirmed through imaging or any history and diagnosis of ossification of the cochlea, (ii) Any abnormality in electrode position as identified through imaging.

A sample size of eight participants was derived from the effect size (Cohen’s *d* of 1.03) from a similar study by Di Maro et al.,^13^ which examined speech reception threshold under pre- and post-ABF methods with a sample size of 10 participants. The mean values from their study^13^ (M1 = 61.25, M2 = 51.25, SD1 = 10.93, SD2 = 3.53), where M1 = Mean value of speech reception threshold of pre-test, M2 = Mean value of speech reception threshold of post-test, SD1 = Standard deviation value of speech reception threshold of pre-test, and SD2 = Standard deviation value of speech reception threshold of post-test, were used. This effect size was converted to eta eta-squared value by the formula (partialη^2^ = Cohen'sd^2^ × N/Cohen'sd^2^ × N + N−1),^17^ which gave the value of partialη^2^ of 0.54. The current study equipped nine CI ears (numbered CI-1 to CI-10) from eight participants with ABF maps.

### Preparation, pilot tests, and validation of Subjective rating test materials

The preparation of subjective rating test materials, their validation, and the pilot testing are detailed in the supplementary test material preparation document. Three normal-hearing listeners validated the materials developed, and then it was piloted on three experienced adult CI users. Testing was conducted in a sound-treated audiology booth where participants were seated one meter away from the loudspeaker at 0 ° azimuth at 30 dB Sensation Level (SL) re: average aided thresholds in the frequency octaves of 500 Hz to 2 kHz to ensure the most comfortable listening range. The pilot tests aimed to select the best stimuli for the field study.

### Main field testing

The main field test was conducted with validated passages for subjective rating and a 35-item CIQOL questionnaire^16^ at two time points: pre- and post-ABF, as further described below.

All participants were tested using their own speech processors with the commonly used speech processor settings. Pre-ABF, participants were tested using their CBF maps. The CBF results served as the baseline reference and were considered the control compared to the ABF map results because participants had more experience with the CBF map. Then, the ABF map was created, and outcome measures were repeated six months post-ABF.

### Aided thresholds

All participants must have their aided thresholds at octave frequencies 250 Hz, 1000 Hz, 2000 Hz, and 4000 Hz within 20–40 dB HL for outcome measure assessment. The test material was presented through a loudspeaker at 30 dB SL (i.e., 30 dB above their aided thresholds at the three-frequency average of 500 Hz, 1000 Hz, and 2000 Hz).

### Outcome measures

#### Subjective ratings

The recorded passage chosen from the pilot testing was used, and the test setup and presentation level were similar to pilot testing (details in the supplementary document). A 5-point Likert scale was used to elicit subjective ratings for the following domains: (i) Listening comfort, (ii) Speech understanding, and (iii) Map preference (Table 1).

**Table 1:** Subjective rating rubric scale.

Domains	Scale				
	1	2	3	4	5
Listening comfort	Extremely poor listening comfort	Poor listening comfort	Neither poor nor good listening comfort	Good listening comfort	Extremely good listening comfort
Speech understanding	Extremely poor speech understanding (Can understand 1%‒20% of the passage)	Poor speech understanding (Can understand 21%‒40% of the passage)	Neither poor nor good speech understanding (Can understand 41%‒60% of the passage)	Good speech understanding (Can understand 61%‒80% of the passage)	Extremely good speech understanding (Can understand 81%‒100% of the passage)
Subjective preference of the fitting mode	Extremely not preferred (1%‒20%)	Considerably not preferred (21%‒40%)	Slightly preferred (41%‒60%)	Preferred (61%–80%)	Extremely preferred (81%‒100%)

#### Cochlear Implant Quality of Life (CIQOL-35)

Participants were asked to rate the 35-item CIQOL questionnaire,^16^ which was prepared using Google Forms, a cloud-based survey software, at baseline (when using the CBF map) and after six months of using the ABF map. The CIQOL-35 instrument has six independent domains and measures the quality of life of CI users using a 5-point Likert scale: 1 = Never, 2 = Rarely, 3 = Sometimes, 4 = Often, and 5 = Always. The six profile domains include communication, emotional, entertainment, environment, listening effort, and social aspects of life.

### ABF method setup and outcome measures

**Fig. 1:**
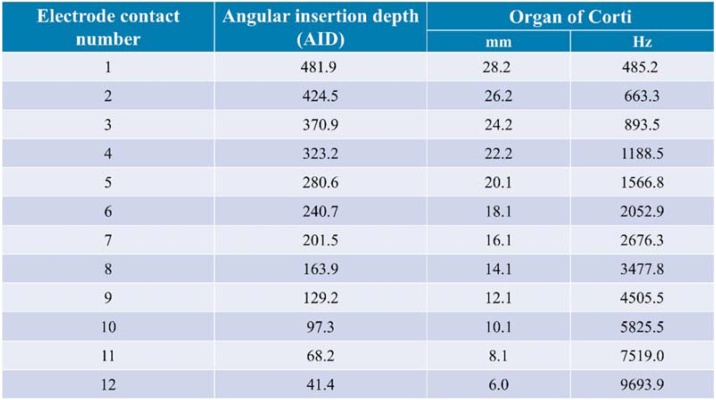
The information generated by the Otoplan® software after electrode point identification for one of the participants in this study. The electrodes’ positions were detected after defining the cochlear anatomy from different views, and subsequently, the angular insertion depth (degrees) was defined for each electrode. The electrode lengths (mm) and center frequencies (Hz) for all contact points are defined, respectively.

Participants underwent post-operative High-Resolution Computed Tomography (HRCT) scans, and these HRCT-CI images were analyzed by a CI radiologist using the Otoplan®, where each electrode's anatomical contact was detected, and respective angular insertions and center frequencies were defined as shown in Fig. 1 for one of the participants. The information gathered was transferred to the Maestro 9.0 (MED-EL, Innsbruck, Austria) CI fitting software. Subsequently, the ABF programming was done in the Maestro interface, where the anatomy-based Organ of Corti (OC) option was selected as the frequency distribution. For participants with Sonnet and Rondo 2 processors, the ABF frequency allocation table was entered manually in their own earlier-generation speech processors after creating it using Sonnet 2 or Rondo 3. Participants with the latest audio processors (Sonnet 2 or Rondo 3) received the ABF map directly in their processors. Further adjustments were made to ensure participants could accept the new experimental ABF map and use it throughout the 6-month study period.

The same outcome measures were assessed after six months of ABF acclimatization. To avoid the participation of non-test ears, the hearing aids were taken off for bimodal users. For bilateral CI processors, one CI was detached during the assessment, and the assessment was done separately on separate occasions. Finally, the attitude towards the ABF map was obtained.

### Statistical analysis

The data was presented uniformly in a tabular format for the analysis. Data confidentiality was assured by coding the participants and protecting them with a password accessed by the principal investigator only. The results were analyzed using SPSS version 26. A paired *t*-test compared the CIQOL-35 ratings, a Wilcoxon Signed rank test compared the subjective ratings scores and a Spearman correlation was employed to determine the correlation between the improved speech perception scores with the improved CIQOL-35 domains. Since the effect size provides extra information for smaller sample size studies,^16^ the effect sizes were included in the results. Corrections of Cohen’s *d* for a small sample size were reported accordingly with the interpretation of 0.2 as a small effect, 0.5 as a medium effect, and 0.8 as a large effect size.^18^

## Results

### CI-demographics

A total of 21 CI users were approached to participate out of which eight CI users consented to participate. A total of nine CI ears were examined from eight participants (i.e., seven unilateral CI and one bilateral CI). Their mean chronological age was 33.6 ± 9.8 years, range (18–50) years, with the average overall CI hearing experience of 3.2 ± 1.2 years, range (1.1–4.1) years. Other information related to the participants is tabulated in Table 2.

**Table 2:** Participants' demographic and CI details.

Participants	CI ears	Age (years)	Gender	CI experience (years)	Speech processor	Active channels	Type of electrodes	AID (^o^)	Hearing Loss onset
P1	CI-1	35	F	1.66	Sonnet	11	FLEX28	414.9	Postlingual
P2	CI-2	36	M	2.33	Rondo 2	12	Standard 31.5 mm	532.3	Postlingual
P3	CI-3	38	F	3.5	Sonnet	12	FLEX28	501.2	Postlingual
P4	CI-4	50	F	1.08	Sonnet 2	9	FLEX28	358.2	Postlingual
P5	CI(L)-5	34	M	4.08	Rondo 2 (L & R)	12	FLEX28	536.6	Postlingual
	CI(R)-9					12	FLEX28	493.3	
P6	CI-6	19	M	4.41	Rondo 3	12	FLEX28	454.0	Prelingual
P7	CI-7	39	M	4	Rondo 2	12	FLEX28	488.8	Postlingual
P8	CI-8	18	F	3.66	Sonnet 2	11	Standard 31.5 mm	458.5	Prelingual

*CI, Cochlear Implant; L, Left; R, Right; M, Male; F, Female; AID, Angular Insertion Depth.

### Center frequency shifts in semitones

The actual place pitch shift detected from imaging was the differences in the center frequencies of the frequency allocation tables between the ABF and the CBF maps. Some participants complained about strange, intolerable sounds using the default ABF map generated by the Maestro fitting software. As a remedy, the frequency band of the first electrode contact was adjusted to less than or equal to 250 Hz, as suggested by a similar study.^14^ The frequency shifts were converted to semitones using the following formula:^19^ N=12×log₂(f₂/f₁); where N = frequency shift in semitone, f_2_ = ABF center frequency; f_1_ = CBF center frequency.

The frequency shifts varied across electrodes, ranging from 0.46 semitones (0.04 octave) at (E12) for CI-2 to 23.94 semitones (1.99 octaves) at (E1) for CI-4, as shown in Table 3.

**Table 3:** The magnitude of FPM for all participating ears and respective channels in semitones.

CI-ears	CI electrodes											
	1	2	3	4	5	6	7	8	9	10	11	12
1	3.0	7.3	8.4	8.3	8.6	7.5	5.6	4.5	3.7	2.2	0.6	
2	5.7	9.1	10.8	10.5	9.4	8.3	7.2	5.9	4.5	3.1	1.8	0.5
3	4.4	7.5	8.5	8.2	7.8	8.6	9.1	9.7	9.8	11.1	3.0	−0.8
4	23.9	23.6	20.7	17.8	15.0	12.8	9.7	5.9	1.8			
5	6.0	9.4	9.9	9.3	8.4	8.2	8.7	8.0	6.2	4.4	2.6	0.7
6	12.8	20.4	21.8	20.5	19.0	16.7	13.8	11.0	8.5	5.9	3.5	0.9
7	11.4	18.0	17.4	13.9	10.9	8.6	7.1	6.1	4.6	3.2	1.9	0.5
8	11.2	13.7	14.3	14.9	14.2	13.1	11.3	9.2	7.1	4.9	1.8	
9	3.4	5.9	7.4	7.8	8.2	7.6	6.8	5.7	4.6	3.4	2.1	0.6

### Outcome measures

#### Subjective ratings

Wilcoxon signed rank test revealed the significance in the mean difference with a medium effect size across all three domains, as shown in Table 4.

**Table 4:** Subjective rating scores.

Outcome Measures	Raw scores (pre)	Raw scores (post)	The Sum of the Mean Rank	Z	Sig.	*r*
Listening Comfort	31	39	15	2.1	0.03*	**0.5**
Speech Understanding	25	38	28	2.4	0.01*	**0.6**
Preferred Map	25	39	21	2.7	0.024*	**0.6**

#### Cochlear Implant Quality of Life -35 (CIQOL-35)

The paired-t results showed that the overall CIQOL-35 scores significantly improved after using the ABF map for six months. The entertainment domain showed a significant increase in the mean difference (p < 0.05). In contrast, all the other domains revealed a slightly improved mean difference except for the emotional domain, which remained the same as tabulated in Table 5.

**Table 5:** CIQoL-35 scores.

Domain	Map	Mean	SD	t-value	Sig. value	Hedges G
Overall scores (CI-QOL-35)	ABF	132.8	14.8	2.9	0.02*	**0.3**
	CBF	128.3	15.7			
Communication	ABF	35.7	7.0	1.0	0.33	0.12
	CBF	34.8	6.6			
Emotional	ABF	18.6	4.7	0.0	1.00	0
	CBF	18.6	3.7			
Entertainment	**ABF**	**20.1**	**3.9**	3.6	0.007*	**0.40**
	**CBF**	**18.8**	**4.3**			
Environment	ABF	21	2.8	0.4	0.67	0.11
	CBF	20.6	4.0			
Listening effort	ABF	19.8	2.3	0.9	0.36	0.4
	CBF	18.9	2.2			
Social	ABF	17.7	3.2	1.5	0.16	0.26
	CBF	16.8	3.3			

#### Attitude towards the ABF map

Participants were asked to fill out a feedback form at the study's end to summarize their views regarding the ABF MAP and their preferred fitting mode before winding up the project, as reported in Table 6. Most of the participants (87.5%) selected the ABF MAP. Six participants (75%) agreed 100% that the ABF MAP perceptually sounded better than CBF and was worth trying despite the preceding stage requirements.

**Table 6:** Attitude towards ABF map.

Points	Responses from 8 Participants		
How often will you use ABF MAP in the future?	All the time	Sometimes	Not at all
	(87.5%)	(12.5%)	0
Will you recommend ABF MAP to other CI users?	To all CI users	To those not happy with CBF	Not at all
	(50%)	(50%)	0
Do you feel that the ABF MAP is worth trying despite extra investigations like CT scans and extra mapping?	Yes agree 100%	Partly 50%	Not at all
	(75%)	(25%)	0
Overall, how would you rate the ABF MAP as compared to your old (CBF) MAP regarding your listening experience?	Better	Average	Poorer
	(75%)	(25%)	0

All of the ratings were improved after using ABF for six months. This could be due to improved speech perception scores, both vowel and consonant perception in quiet and in the presence of noise. A brief data concerning speech perception at baseline for CBF and after ABF use is detailed in Table 7, and the full detail can be found in a previous paper from the same study setting by Wangchuk et al.^15^

**Table 7:** Speech perception scores (%).

	Consonant perception in quiet (%)		Vowel perception in Quiet (%)		Consonant perception in noise (%)		Vowel perception in noise (%)	
CI-Ears	CBF	ABF	CBF	ABF	CBF	ABF	CBF	ABF
1	29.6	50	92.6	100	9.3	49.1	80.6	100
2	13.8	25.9	42.6	97.2	6.5	19.4	41.7	93.5
3	7.4	33.3	44.4	97.2	11.1	21.2	35.1	94.4
4	12	36.1	76.8	98.1	3.7	27.8	58.3	98.1
5	28.7	37.0	73.1	88.9	12	22.2	52.8	85.1
6	31.4	42.6	95.3	94.4	27.8	35.9	88.9	94.4
7	13.8	15.7	31.5	48.1	5.5	8.3	18.5	37.0
8	2.7	39.8	34.3	91.7	14.8	27.8	71.3	88.9
9	10.1	41.6	66.7	90.7	3.7	34.3	50	90.7

#### Correlation between the speech perception scores with the CIQOL-35 domains

Spearman’s correlation analyses revealed a strong correlation (*r* = 0.77, p = 0.01) between the entertainment domain and vowel identification in quiet. These findings suggest that better vowel recognition in quiet is strongly linked with improved enjoyment of entertainment activities. Communication and Social also show moderate-to-strong positive trends, although not statistically significant, possibly due to the small sample size (Table 8).

**Table 8:** Correlation between improved speech perception and CIQOL domains.

	Sig	Communication	Emotion	Entertainment	Environment	Listening effort	Social
Vowel in quiet	*r*	0.616	0.286	0.772*	−0.111	−0.496	0.483
	p	0.078	0.456	**0.015**	0.777	0.175	0.188
Vowel in noise	*r*	0.180	0.151	0.634	0.128	−0.291	0.345
	p	0.644	0.698	0.067	0.743	0.448	0.363
Consonant in quiet	*r*	0.051	0.370	0.109	0.187	−0.009	0.621
	p	0.896	0.327	0.780	0.629	0.983	0.074
Consonant in noise	*r*	−0.254	−0.051	0.025	0.588	0.479	0.278
	p	0.509	0.897	0.949	0.096	0.193	0.468

## Discussion

This study assessed the benefits of the ABF over the CBF maps among eight experienced adult CI users over six months. Outcome measures include (i) Subjective ratings in speech understanding, listening comfort, and map preference, and (ii) QOL.

Wilcoxon signed rank test revealed a significant mean difference with a medium effect size across all three domains concerning subjective ratings. None of the three earlier published studies by Di Maro et al.^13^ and Kurz et al.,^14^^,^^20^ involving ABF, reported this aspect for comparison. However, a recent study by Lassaletta et al.^12^ reported insignificant improvement in speech reception and speech discrimination outcomes with the ABF map after nine months of usage. However, the same study reported that most patients (seven out of eight) preferred the ABF map at the end of their study.^12^

The subjective rating scores improved with the ABF map in the current study, indicating the benefits of various auditory experiences. A recent study involving ten experienced adult CI users compared the self-perceived sound quality between the CBF and three-month post-ABF maps, in which participants reported similar outcomes.^20^ It could be that 3-months is too short to judge the difference in sound quality of the two maps. In another study by Lasaletta et al.,^12^ seven out of eight participants preferred the ABF map, although speech understanding was not significantly improved. This is also consistent with our findings, as listening comfort and preferred map ratings were markedly improved after six months of ABF use. The ABF map over time was also found to improve the perception of vowels and even voiced consonants,^15^ and these improvements in speech perception could have influenced their favouring of the ABF map during their subjective ratings at six months of ABF experience. The results could be because of the advantage that ABF possessed, as the map follows the Greenwood function.

The overall CIQOL-35 scores significantly improved after using the ABF map for six months, p < 0.05, with the entertainment domain revealing a significant improvement. This could be because some participants personally reported that they could enjoy television and movies with the ABF map compared to their old CBF map. The CIQOL-35 scores in all domains showed a trend of improved scores, although they were statistically insignificant. The most significant domain change rated as entertainment suggests that these participants could have rated more positive experiences with the ABF over time due to their better perception of speech and enjoyment of entertainment. These results were consistent with the better self-perceived sound quality rating and its effect, as reported in a recently published study of ABF.^20^

It was also found in this study that there was a significant and strong correlation between the entertainment domain and vowel perception in quiet. The observed association suggests that patients with better vowel perception tend to experience a more natural and pleasant auditory perception during exposure to TV, radio, or music. This highlights the importance of optimizing the frequency-to-place alignment within the cochlear implant system. Vowels are primary carriers of temporal and prosodic information critical for rhythm, emotional content, and natural sound quality in both speech and music.^21^^,^^22^ Earlier studies revealed that a reduction in subjective music and entertainment enjoyment is a common complaint among CI users, which is significantly correlated with self-assessed hearing ability and quality of life, even when standard speech scores are high^23^^,^^24^ ABF and individualized frequency mapping have been shown to enhance the perception of critical acoustic features (including vowel formants), leading to improved speech understanding in various listening conditions, superior sound quality, and greater music appreciation.^15^^,^^25^ This body of work support our findings on the association between vowel perception and the entertainment domain, providing a robust rationale for implementing individualized programming strategies like ABF to enhance real-world listening experiences beyond simple speech perception tests.

Although there was a trend of improved QoL and subjective ratings for all domains, the variations across participants prevailed. The variation in the perceived improved QoL with ABF could also be due to the variation in the CI electrode array design, leading to variations in the degree of FPM and its impact on CI-specific QoL.^10^ Our findings, however, contradict those reported by Lambriks et al.,^26^ who fitted 14 adult CI recipients with image-guided fitting maps and examined their speech perception in quiet and noise, sound quality, and listening effort with these maps after 12 weeks of implant experience. The imaging-based fitting was rated as significantly dull/damped (+3-weeks: p = 0.01, +12-weeks: p = 0.01) and unclear/blurry (+3-weeks: p = 0.01, +12-weeks: p = 0.02) on sound quality assessment. Comparing listening effort between the imaging-based and standard fitting revealed no significant differences after correction for multiple comparisons.^19^ However, the acclimatization effect could have played a role in the present study 87.5%. of the participants showed a positive attitude towards ABF and wanted to continue with the map after completion of the 6-month ABF study. Six out of eight participants agreed that it was worth trying despite the requirements of the preparatory stage (i.e., the need to go through the CT scan prior to ABF).

The under-representation of data due to a relatively small sample size is an underlying limitation in the present study. However, the results are likely generalizable at least among experienced, adult CI users, due to a sufficient acclimatization period, as studies suggest a minimum of six months for complete acclimatization for the new map for CI users.^27^

## Conclusion

In this sample, the subjective ratings of the map preference favoured the ABF over the CBF method after six months of ABF acclimatization. This leads to a hypothesis regarding the possibility that the amount of FPM in their previous CBF maps accounted for the less comfortable maps, leading to poorer speech understanding and listening comfort. Coinciding with this finding, the overall rating for the QoL also showed improved scores, with the entertainment domain being the most significant. Overall, their attitude towards the ABF map was very high. However, more data are necessary to generalize the benefits of the ABF. The present study proposed that the ABF map is an alternative that CI audiologists should consider to improve auditory perceptions, at least among adult CI users.

## Level of evidence

How common is the problem? Level 3.

Is this diagnostic or monitoring test accurate? Level 3.

What will happen if we do not add a therapy? Level 4.

What are the treatment harms? Level 5.

Is this early detection worthwhile? NA.

## ORCID ID

Chong Foong Yen: 0000-0003-4964-4694

Faizah Mohd Zaki: 0000-0002-7142-3794

Asma Abdullah: 0000-0002-0103-8858

## Dual first authorship request

Special request to consider dual first authorship as sequenced (Pelden Wangchuk, Cila Umat).

## Ethical approval

This study will comply with ethical principles outlined in the Declaration of Helsinki and Malaysian Good Clinical Practice Guidelines. The Universiti Kebangsaan Malaysia (UKM) has approved this research plan through the UKM Research Ethical Committee dated 28 July 2022 with the reference number UKM PPI/111/8/JEP-2022-392.

## Funding

The MED-EL company, especially MED-EL (Malaysia) supported this study through training, technical support, and funding (NN-2022-022) to ensure the success of this project.

## Data availability statement

The authors declare that all data are available in repository.

## Declaration of competing interest

The authors declare no conflicts of interest.
