# Supplementary material for: Assessing the quality of life and subjective ratings of the anatomy-based fitting map among experienced, adult cochlear implant users
Source: Braz J Otorhinolaryngol. 2026 Mar 28;92(3):101807. doi: 10.1016/j.bjorl.2026.101807 (PMC13062517; doi:10.1016/j.bjorl.2026.101807)
Supplement: Supplementary file 1 [file mmc1.docx]

Phase 1. Test material preparation and stimulus development

The test materials to be used in this study were meant for different specific objectives. Three test materials were developed as described later in this section. Audacity and Praat software were required for recording and editing sound during the test stimulus development phase. Audacity and Praat is an easy-to-use, free, and open-source software that can perform multi-track audio editor and a recorder for computer operating systems. A calibration tone in Audacity was generated at 1000 Hz equalized at the same root mean square (RMS) level of 60dB and used as the calibration tone to equalize passages and the recorded nonsense syllables during the field test. White noise was generated using the Audacity program and used as the background noise during vowel identification in the noise test.

Speech perception

Nonsense Syllable

Nonsense syllables comprising of consonant-vowel-consonant (CVC) format were prepared. Despite the availability of numerous validated and reliable speech test materials, nonsense syllables were used because the primary aim of the study was to assess auditory perception and not necessarily meaning. Nonsense CVC syllables were found to yield poorer recognition performance compared to meaningful CVC syllables suggesting that the lexical content may add to the quick judgment and guessing of the words which may impact the actual auditory perception evaluation. The study also suggested that the use of nonsense CVC syllables provides a way to assess speech recognition while potentially lessening the effects of lexical content on auditory perception performance (Findlen & Roup, 2011). Vowels /a/, /i/, /u/ were paired with initial voiced and voiceless plosive consonants and final nasal consonant /n/, as shown in Table 3‑1. Vowels were used as they have periodic and repetitive waveforms with high energy which refines in both perceptual and productive aspects essential to speech intelligibility (Kent & Rountrey, 2020). Moreover, in the experimental results of an assessment of segmental contributions to speech perception by CI listeners, results showed that segmental interruption had a significant influence on CI speech perception, and vowels were found to represent more dynamic perceptual perception than consonants (Chen & Hu, 2019).

Table 3.1 Vowel identification test material

| Initial consonants | Vowels | Final consonant | Stimulus set |
| --- | --- | --- | --- |
| Bilabial voiced plosive /b/ | /a/  /i/  /u/ | /n/ | /ban/, /bin/, /bun/ |
| Bilabial voiceless plosive /p/ |  |  | /pan/, /pin/, /pun/ |
| Dental voiced plosive /d/ |  |  | /dan/, /din/, /dun/ |
| Dental voiceless plosive /t/ |  |  | /tan/, /tin/, /tun/ |
| Velar voiced plosive /g/ |  |  | /gan/, /gin/, /gun/ |
| Velar voiceless plosive /k/ |  |  | /kan/, /kin/, kun/ |

Three voiced and three voiceless plosives were set as initial sound which was combined with three different vowels. The nonsense syllable tot up to 18 for each set. The list was randomized into three sets summing up to 54 samples.

Recording of the nonsense syllables:

The recording was done in at Audiology suite inside the sound-treated audiometric booth. The recording hardware included the Audio-Technica AT2020 cardioid condenser microphone (Audio-Technica U.S Inc, Stow, OH, USA) connected to a laptop via the Focusrite Scarlett Solo 2nd generation USB audio interface. The recording software was Audacity version 3.1.3. Three sets containing 54 CVC tokens were recorded by a native Malay male and a female speaker. The CVC tokens were recorded at a sampling rate of 44,100 Hz and each CVC recording was preceded by a carrier phrase while recording to ensure the adequate sampling of the token. The recorded tokens were stored with a depth of 32-bit quantization WAV files for offline analysis and further processing.

Visual and auditory inspections were conducted on the acoustical waveforms and spectrograms of the recorded nonsense syllables tokens (n=108) and passages (n=4) that were generated using the PRAAT software (version 6.2.10) to complete the following process as described below.

Token extraction and equalization:

The sound file was imported into the PRAAT software. The following tasks of sound extraction and edition were carried out.

1. Speech syllable was transcribed in PRAAT, by annotating to the text grid and determining the boundaries of each nonsense word within the recordings with carrier phrases as shown in Figure 3‑1. This process also served as the reference for visualizing the sequence of the nonsense syllable in each recording. During the sound extraction, the following steps were taken.
   1. The beginning of the initial consonant in the CVC was defined after identifying the release burst in the spectrogram. The extraction of the sound was done by moving the cursor approximately 50ms backward to preserve the naturalness of the extracted token sounds, as illustrated in Figure 3‑2 and Figure 3‑3.
   2. The end of the final consonant in the CVC was defined when the spectrogram showed white space continuously. The extraction of CVC length approximately included 300ms from the end of the final consonant to increase the smooth cessation of the token.

The CVC extracted was rechecked by carefully zooming and ensuring it was free from idiosyncrasy elements such as unclear burst waveforms for consonant plosives and unintended noise or click sounds and uneven tone contour as shown by voiceless consonant syllable /tin/ in Figure 3‑4.

1. The above-extracted sound was saved as a 32-bit WAV file using PRAAT software. The extracted sound samples were further processed in the Audacity to equalize the root-mean-squared (RMS) voltage level at a uniform 60 dB. This step was done to match the 1000Hz calibration tone which was also equalized at the same RMS level of 60dB.
2. The final equalized tokens were concatenated into word strings with a 500ms interstimulus interval (D’Alessandro et al., 2018; Pisoni, 1975). White noise was generated and equalized using the Audacity program which was used as background noise during vowel identification in noise. These were the final stimuli set for the pilot testing and selection of best exemplars to be included in main field testing.

Examples:

Transcribing the tokens from recording


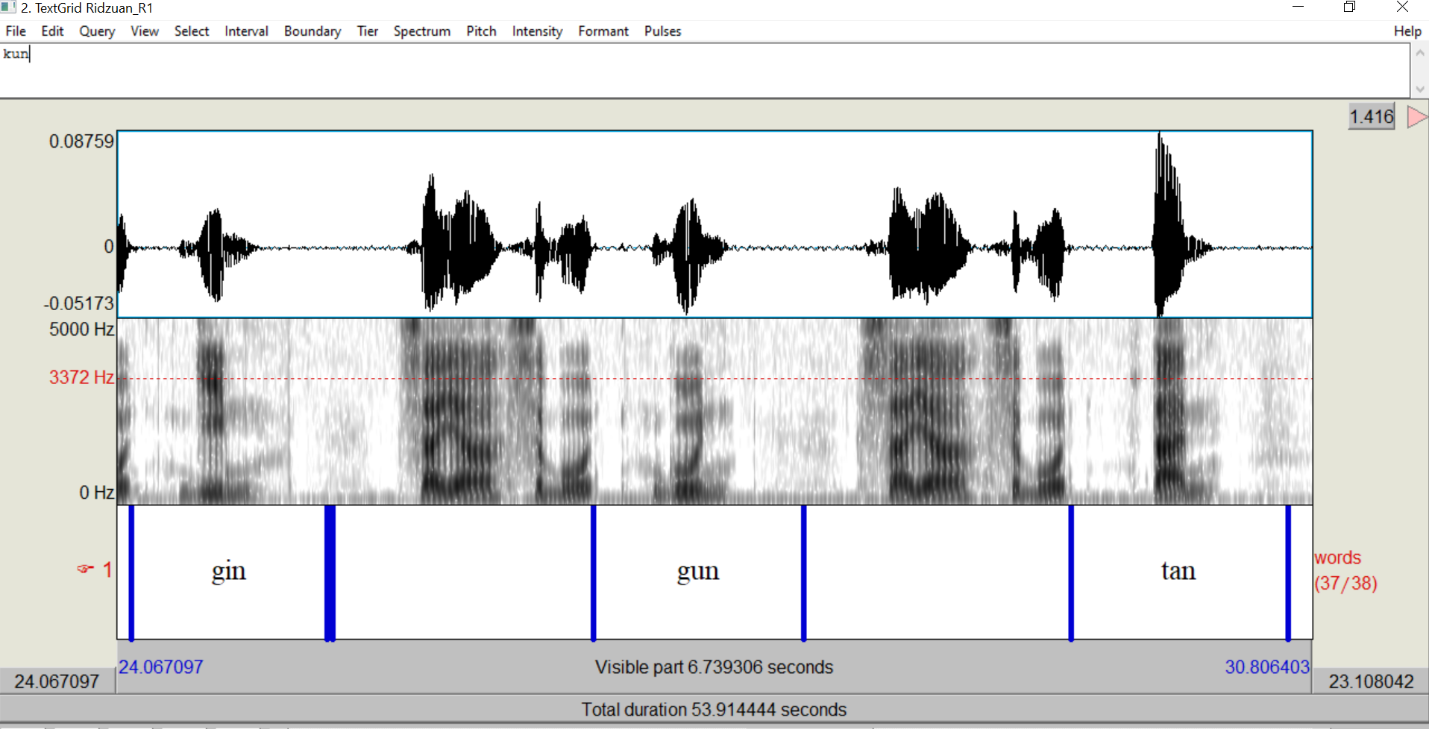


Figure 3.1 text transcribed to the raw recording string.

Voiced plosives /ban/ extracted after text transcription.

1. Ban with carrier time:

The carrier time was constantly defined at 50ms before the onset of the burst. This was to assure the preservation of the release burst of the plosive consonants and to promote a clearer presentation of the token. The endpoint of the token was defined when the waveform diminished. The selected portion in the figure is the sample extract of /bin/.


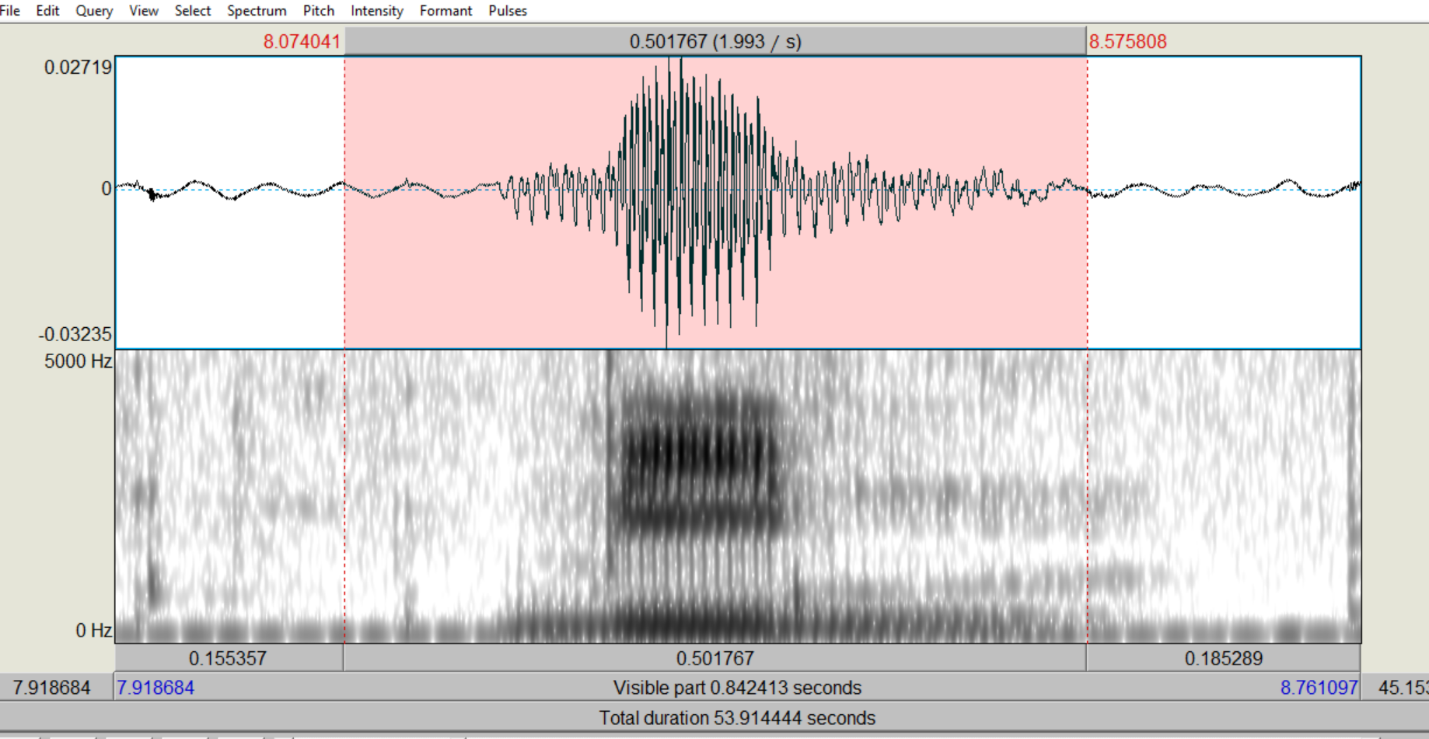


Figure 3.2 Extraction of token /ban/ after defining the time frame.


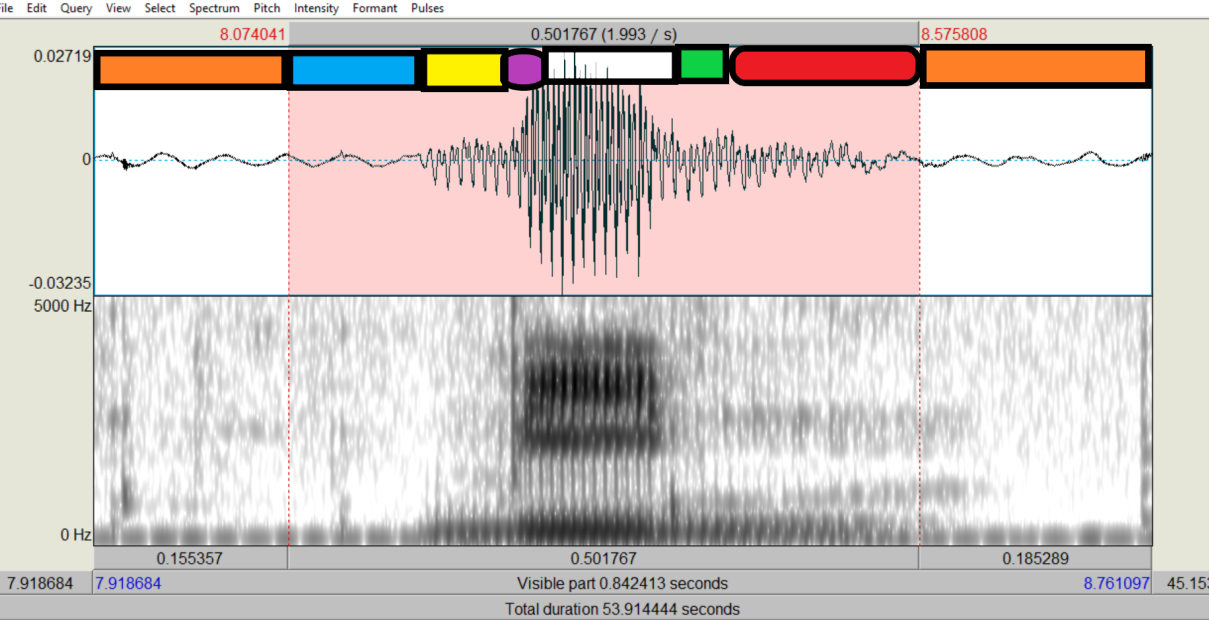


Figure 3.3 Spectrogram analysis of token /ban/ explained by color coding.

1. blue: carrier time end
2. Yellow: release of the burst /b/
3. Purple: aspiration, delay of onset of the voicing
4. White: finally voicing
5. Green: onset of nasal sound
6. Red: Defining the end of the extract
7. Brown: two extreme sides of brown are trimmed out of the extract.

Voiceless plosives


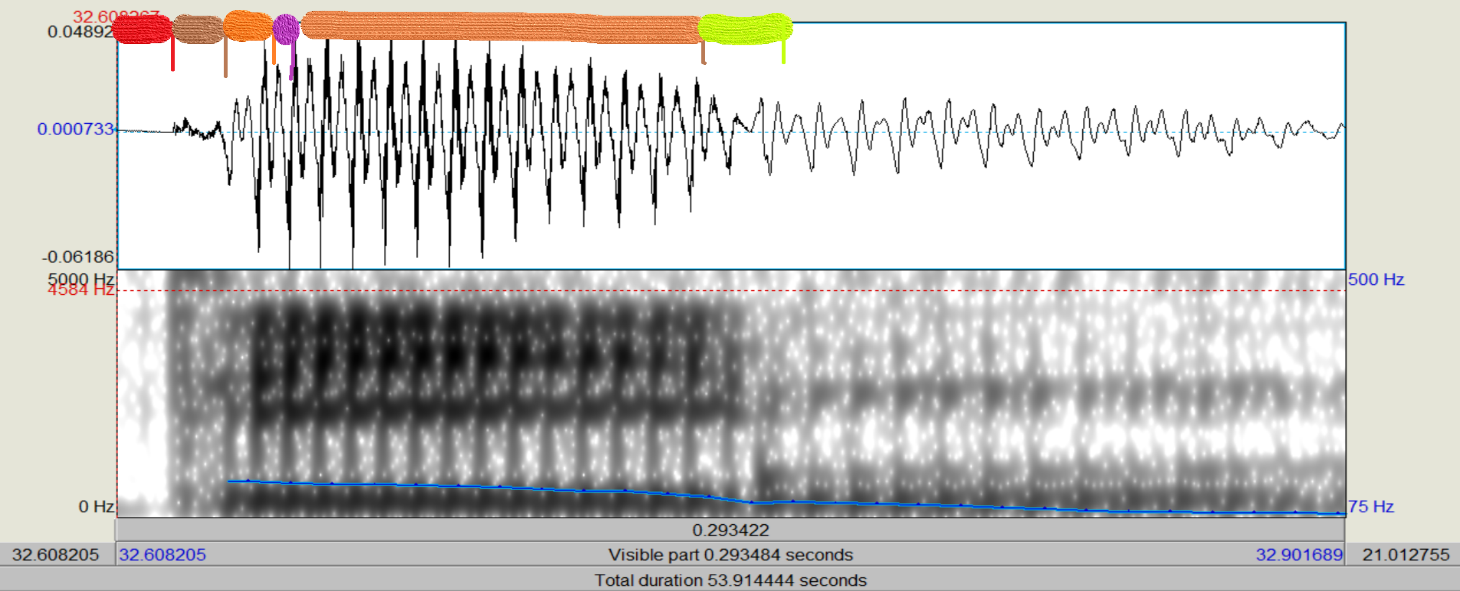


Figure 3.4 spectrogram analysis of token /ban/ explained by colour coding.

1. Red: carrier time end. The length of silence extracted from this section is 50ms.
2. Brown: the medial phase of /t/ silence (this is present for the unvoiced consonants)
3. orange: release of the burst /t/ consonant
4. purple: aspiration, delay of onset of the voicing
5. light brown: finally voicing /i/
6. yellow: onset of a nasal sound /n/.
7. The endpoint is defined when the waveform diminishes.

After analysis and extraction, a total of three tokens were excluded as the tokens did not meet the extraction criteria. A total of 105 sample tokens were set for the pilot testing. The spectrograms of the final tokens that were set for the pilot are shown in the figure below.


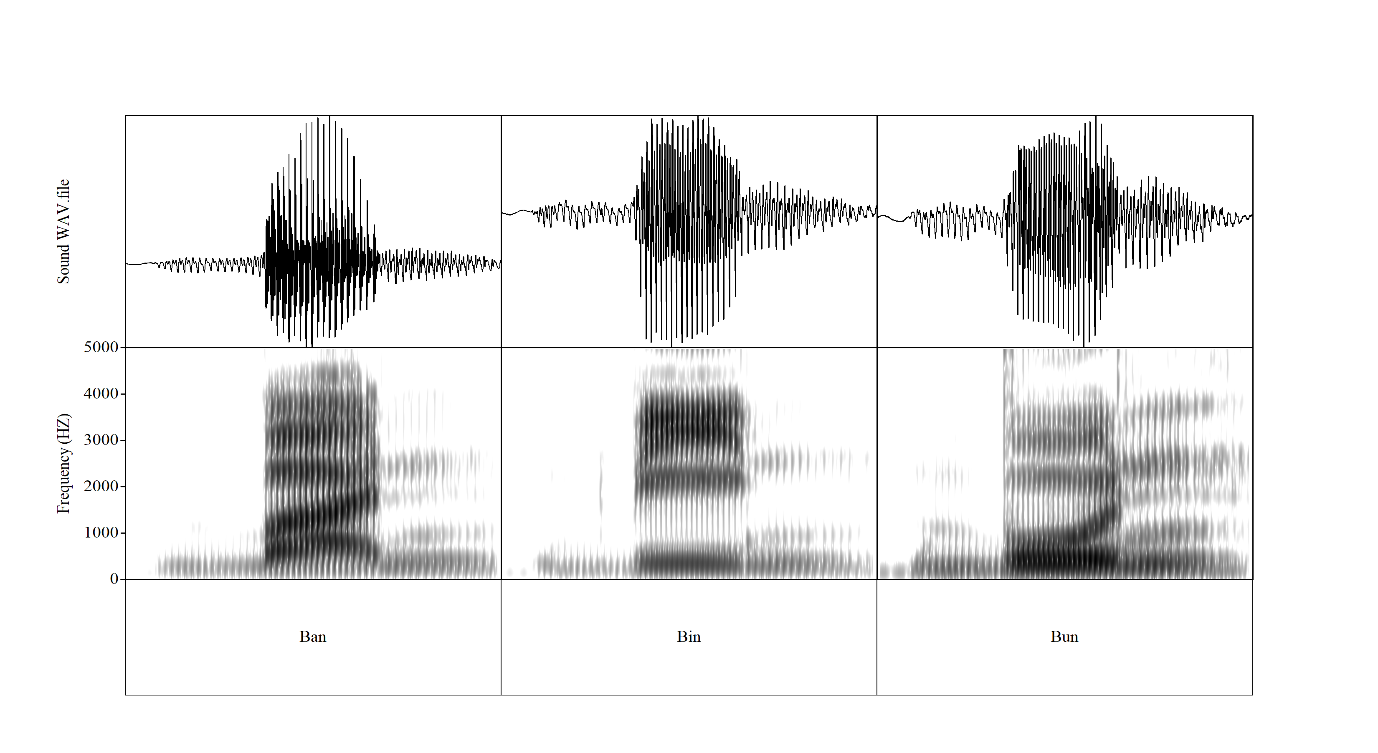


Figure 3.5 Sample of sound wave and spectrogram used for the pilot test.

Subjective ratings

Two types of materials were prepared: - (i) a recorded passage. (ii) a Visual Analogue Scale (VAS)

Passage preparation:

A passage narrating a simple story with decent length (100 to 150 words) having familiar words was prepared as a sample. The fairy tale “North Wind and the Sun” in Malay and English language was recorded by both genders with a total of four sample passages as shown in the box below. The whole passage was extracted and equalized with a root mean squared electrical voltage level of 60 dB. The samples were stored in a WAV file. A 1000 Hz calibration tone in Audacity was generated and used as the calibration tone to equalize all two passages during the test. A total of four passages were extracted and equalized for the validation process.

The North Wind and the Sun (English passage)

The North Wind and the Sun were disputing, which was the stronger when a traveller came along wrapped in a warm cloak. They agreed that the one who first succeeded in making the traveller take his cloak off should be considered stronger than the other. Then the North Wind blew as hard as he could, but the more he blew the more closely did the traveller fold his cloak around him, and at last the North Wind gave up the attempt. Then the Sun shone out warmly, and immediately the traveller took off his cloak. And so the North Wind was obliged to confess that the Sun was the stronger of the two.

Angin Utara dan Matahari (Malay passage)

Angin Utara dan Matahari berselisih tentang mana yang lebih kuat apabila seorang pengembara datang berselimut dengan jubah. Mereka bersetuju bahawa orang pertama yang berjaya membuat pengembara itu menanggalkan jubahnya harus dianggap lebih kuat daripada yang lain. Kemudian, Angin Utara bertiup sekuat yang boleh, tetapi semakin dia bertiup, semakin rapat pengembara itu memegang jubahnya; dan akhirnya Angin Utara menyerah kalah. Kemudian, Matahari bersinar dengan hangat dan pengembara itu menanggalkan jubahnya dengan segera. Maka, angin Utara terpaksa mengaku bahawa Matahari adalah yang lebih kuat di antara kedua-duanya.

Visual analogue scale (VAS)

The rubric scale and subjective ratings were prepared differently for the pilot testing and main field testing. This is because the pilot test was aimed at the selection of the passage which had the best representation of fluency, natural expression, and clarity of words whereas the field test aimed to find out the preferred program as shown in Table 3‑2 and Table 3‑3.

Table 3.3 Subjective rating domains used in the pilot testing.

| **Domains** | **Scale** | | | | |
| --- | --- | --- | --- | --- | --- |
|  | 1 | 2 | 3 | 4 | 5 |
| Fluency | Choppy, needed to stop and figure out words. | Stopped now and then to work on a word. | Smooth like a river. Heard every word | Flowed the words with the mood of the story. | Extremely good listening comfort |
| Naturalness in expression | Didn’t change voice at all: monotone | Read in a sing-song way or did not change voice much | Changed their voice to make interesting | Sounded very professional. | Perfect to be a reader on stage. |
| Clarity | Mumbled a lot. Could not understand | Mumbled a bit. Hard to understand all the words. | Pronounced all the words correctly. | There was an extra crispness to the pronunciation | The passage was 100% clear and needed no corrections. |

Table 3.4 Subjective rating rubric used during main field testing.

| Domains | Scale | | | | |
| --- | --- | --- | --- | --- | --- |
|  | 1 | 2 | 3 | 4 | 5 |
| Listening comfort | Extremely poor listening comfort | Poor listening comfort | Neither poor nor good listening comfort | Good listening comfort | Extremely good listening comfort |
| Speech understanding | Extremely poor speech understanding (Can understand 1 - 20% of the passage) | Poor speech understanding (Can understand 21 -40% of the passage) | Neither poor nor good speech understanding (Can understand 41 -60% of the passage) | Good speech understanding (Can understand 61 -80% of the passage) | Extremely good speech understanding (Can understand 81 -100% of the passage) |
| Subjective preference of the fitting mode | Extremely not preferred (1-20%) | Considerably not preferred (21-40%) | Slightly preferred (41 – 60%) | Preferred (61 – 80%) | Extremely preferred (81 -100%) |

Phase 2: validation of test materials and pilot test

The content validation for the test material developed in Phase 1 was done by three normal-hearing adults. After the validation process, the materials were piloted using three experienced adult CI users. Adult CI recipients other than MED-EL CI users were recruited for pilot tests because there were limited ME-DEL CI users and they had to be reserved as the subjects/participants for the main study. The procedures are described in the following section. Since the aim was to evaluate the outcome between the fitting method used as a CI program, residual hearing of the contralateral ear if any was avoided to mitigate any confounding that could arise during testing. Hence, the non-implanted ear was masked, or hearing aids were turned off for the hearing aid users in the contralateral ear. The following tests were conducted during pilot testing.

Speech perception

Three normal hearing and three experienced adult CI users participated in the pilot test. The materials used were 105-nonsense CVC syllables which were recorded and calibrated during the stimulus development phase. Testing was conducted in a sound-treated audiology booth where participants were seated one meter away from the loudspeaker at 0° azimuth. The test stimulus was prepared into six sets (three from male and three from female recordings) and was presented via loudspeakers at 30 dB SL re: aided threshold to ensure the most comfortable listening range. All six sets of stimuli were presented for three repetitions in randomized order. Nonsense syllable perception was assessed by asking the participants to repeat the correct word after each presentation in an open set design. Despite closed-set designs being faster and easier to administer which is commonly used in clinical settings (Black, 1957), the open-set design was used. This is because, in a closed-set test, the probability of obtaining the right answer by chance is high as the answers are limited to the response set provided by the experimenter. On the other hand, open-set design is clueless and assesses the correct perception. The learning effect which is largely coexisting with the closed set approach as explained by Rødvik et al. (2018) was another reason considered. There are other studies suggesting that assessment applying a closed set of spoken word recognition may not be a valid assessment of speech recognition (Clopper et al., 2006). During the assessments, correct responses were scored with one point while no point was allotted for incorrect or no responses.

The testing was performed for three normal hearings first to validate the content and screen out CVC tokens if required. Then the pilot test was run on three adult experienced CI users. The aim here was to select the best tokens and interstimulus interval (ISI) time. The spectrograms of the CVC tokens selected after pilot testing are shown in the figure below in Figure 3.6 and Figure 3.7.


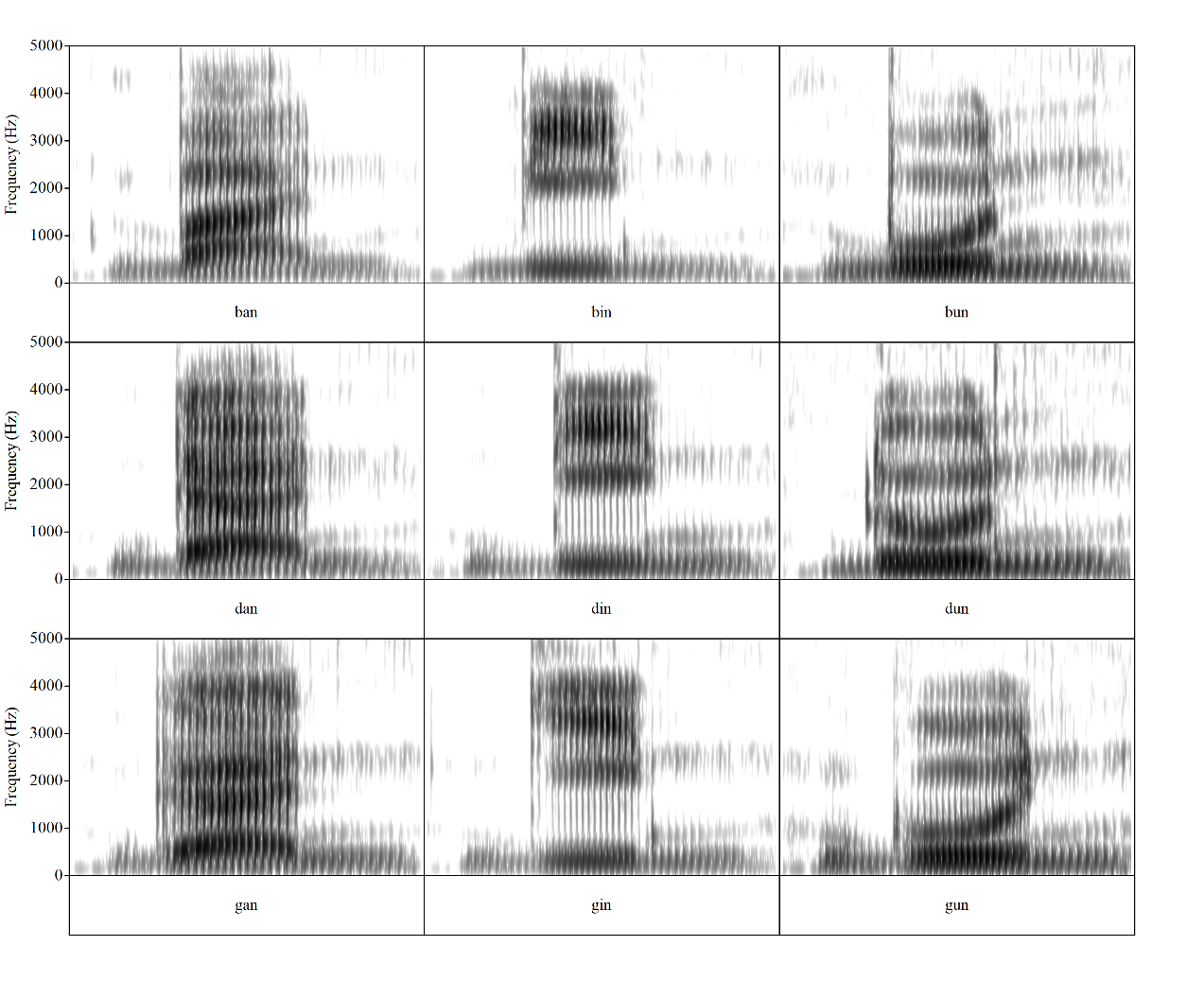


Figure 3.6 Nonsense CVC tokens with voiced consonants


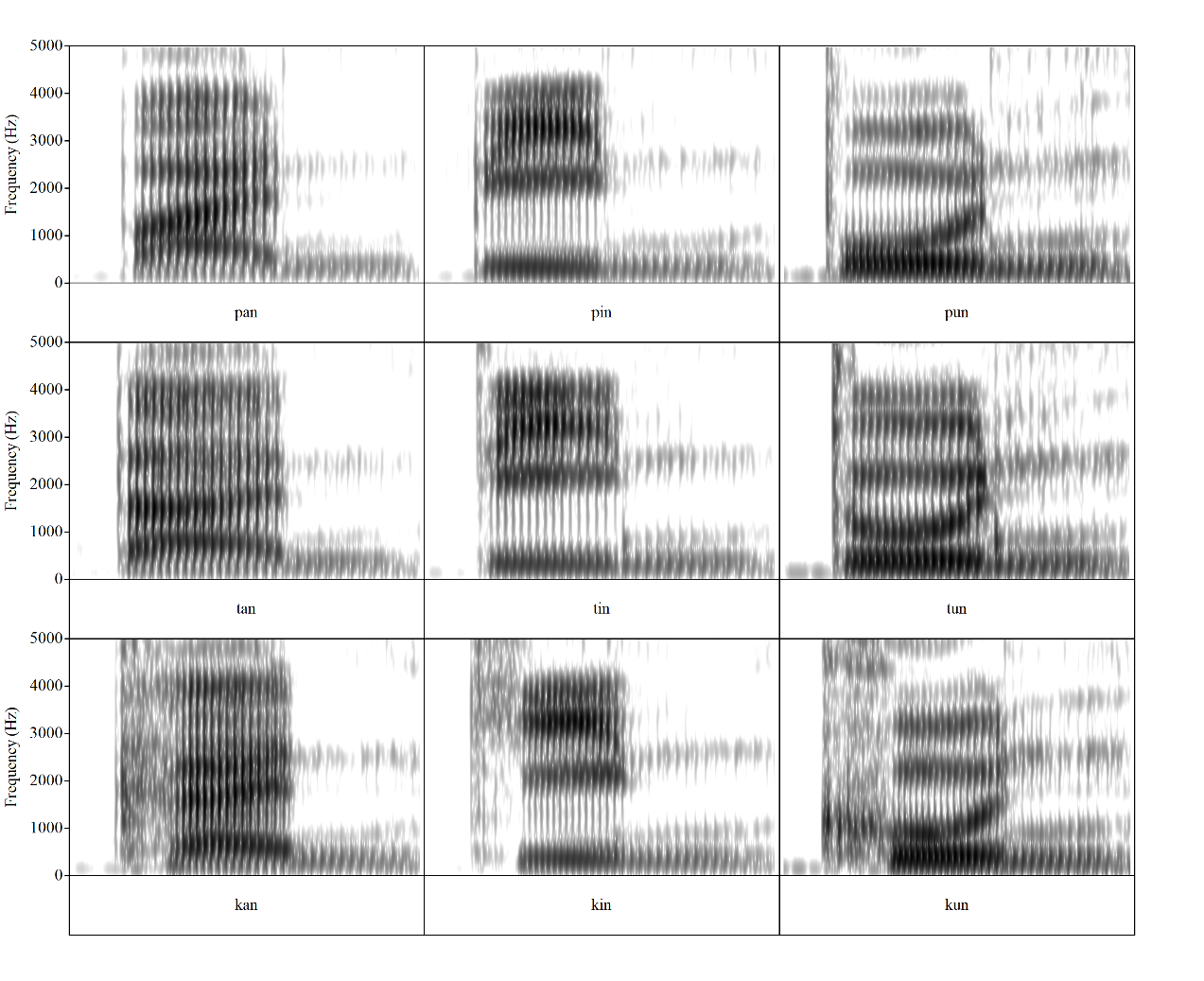


Figure 3.7 Nonsense CVC tokens with voiceless consonants

Subjective ratings

The testing was performed for three normal hearings first to validate the content of the recorded passages. Then three experienced adult CI users were piloted using passages which were refined and calibrated during the material preparation phase. Testing was conducted in a sound-treated audiology booth. Participants were seated one meter from the loudspeaker at 0° azimuth. The recorded passages were played at 30 dB SL. A 5-point Likert scale was used to rate the fluency, naturalness in the expression, and clarity of the recorded passage. The Rubric scale shown in Table 3‑2 was used to rate the passages.
